# Finding a home in the noise: cross-modal impact of anthropogenic vibration on animal search behaviour

**DOI:** 10.1242/bio.041988

**Published:** 2019-07-15

**Authors:** Louise Roberts, Mark E. Laidre

**Affiliations:** 1 Department of Biological Sciences, 78 College Street, Dartmouth College, Hanover, NH 03755, USA; 2 Shoals Marine Laboratory, University of New Hampshire, 8 College Road, Durham, NH 03824, USA

**Keywords:** Animal search behaviour, Anthropogenic noise, Chemical sensing, Cross-modal, Shells, Substrate-borne vibration

## Abstract

Chemical cues and signals enable animals to sense their surroundings over vast distances and find key resources, like food and shelter. However, the use of chemosensory information may be impaired in aquatic habitats by anthropogenic activities, which produce both water-borne sounds and substrate-borne vibrations, potentially affecting not only vibroacoustic sensing but other modalities as well. We attracted marine hermit crabs (*Pagurus acadianus*) in field experiments using a chemical cue indicative of a newly available shell home. We then quantified the number of crabs arriving in control versus impulsive noise conditions. Treatment (control or noise), time (before or after), and the interaction between the two significantly affected the numbers of crabs, with fewer crabs attracted to the chemical cue after noise exposure. The results indicate that noise can affect chemical information use in the marine environment, acting cross-modally to impact chemically-guided search behaviour in free-ranging animals. Broadly, anthropogenic noise and seabed vibration may have profound effects, even on behaviours mediated by other sensory modalities. Hence, the impact of noise should be investigated not only within, but also across sensory modalities.

This article has an associated First Person interview with the first author of the paper.

## Abstract (precis)

**Summary:** Chemical cues enable animals to sense their surroundings and find key resources. Here we show that anthropogenic noise affects a chemically-guided search behaviour by acting cross-modally.

## INTRODUCTION

The environment that an animal inhabits offers a rich array of sensory information spanning visual, auditory, tactile, electrical and chemical modalities (Stevens, 2013). In highly dynamic environments, such as the subtidal, the effectiveness of each sensory mode as an information source fluctuates according to the presence and absence of biotic and abiotic sensory barriers (e.g. light presence, seabed topography, debris), making the use of multiple sensory modes advantageous (Halfwerk and Slabbekoorn, 2015). Chemical cues, in particular, can act across a wide area and disperse rapidly, making them ideal for the transfer of information in marine environments. The ocean consists of a cocktail of chemical cues, which are detected by chemo-receptive organs and allow organisms to interpret their surroundings (Breithaupt and Thiel, 2011). These odorant molecules, which are especially important to organisms with poor visual detection abilities, can provide information regarding shelter, habitats, prey availability, predator proximity, mate location and conspecific status, and as such are fundamental to many marine animals' survival and reproductive success (Hay, 2009).

Humans are contributing additional stimuli, such as light, sound and chemicals to the sensory information that animals have evolved to detect (Stevens, 2013). This anthropogenic sensory pollution can affect animals within the same sensory mode (uni-modally), for example chemical (de la Haye et al., 2012), acoustic (de Jong et al., 2018) and vibratory (Wu and Elias, 2014). Anthropogenic sensory pollution may also affect animals across sensory modes (cross-modally) (Halfwerk and Slabbekoorn, 2015), for example anthropogenic sound affecting the speed of visual signalling (de Jong et al., 2018; Kunc et al., 2014) and response to visual cues (Chan et al., 2010). Interestingly, the effect of anthropogenic noise upon responses mediated by chemical senses has been little studied, though there are indications that noise exposure affects the detection and response to predator-associated cues (Acharya, 1998; Hasan et al., 2018; Morris-Drake et al., 2016). Noise may also affect processes that involve multiple sensory modes (Walsh et al., 2017).

In the marine environment, human activities are adding noise into the underwater system. Water-borne acoustic energy propagates from sources, consisting of a pressure change, and an associated back and forth movement of molecules known as particle motion (Popper and Hawkins, 2017, 2018). Substrate-borne vibrational waves may also propagate through the seabed, particularly when sources directly contact the sediment (Hazelwood et al., 2018; Roberts and Elliott, 2017). Impulsive noise, which involves sudden high pressure and particle motion changes, is of particular interest, since it may increase the likelihood of sensory damage. Examples of impulsive sources include air-guns used to survey the seabed for oil and gas, and pile-driving, a construction technique involving the driving of piles into the seabed during the construction of turbines, platforms, harbours and bridges. Impulsive sound exposures in field conditions may cause behavioural changes, physical damage, mortality and physiological alterations in invertebrates (Fitzgibbon et al., 2017; McCauley et al., 2017), or may have little effect at all (Parry and Gason, 2006). Since invertebrates have received much less research attention than other groups, conclusions are presently extremely difficult to draw (Carroll et al., 2017). Crucially, the potential effects of vibration within the seabed are almost entirely unknown, despite the benthic nature of many marine invertebrates and the direct contact of many anthropogenic sound-producing activities with the seabed (Roberts and Elliott, 2017).

Despite the prevalence of marine invertebrates in the coastal areas where anthropogenic activities often dominate, the effect of anthropogenic noise on animal search behaviours has rarely been explored. One of the most abundant marine invertebrates in coastal areas are hermit crabs (Hazlett, 1981). These animals have an ecological dependency on gastropods for shells (Laidre, 2011), which they use to protect themselves from predators, conspecifics and environmental stresses. Olfactory cues play a key role in shell detection among marine hermit crabs, alerting individuals to the availability of new shells (Rittschof, 1980a; Tricarico et al., 2011; Valdes and Laidre, 2018, 2019). Specifically, peptides released from digested gastropod flesh are highly attractive to marine hermit crabs, a process which can be mimicked by the use of the proteolytic enzyme trypsin (Rittschof, 1980b). Because empty gastropod shells are rare and scattered randomly within a dynamic subtidal environment (Valdes and Laidre, 2018), prompt detection and response to chemical information from newly available shells is particularly important. Any impairment in these responses due to anthropogenic noise could have detrimental fitness consequences.

Here, we tested the effect of an impulsive noise source on the chemically-mediated shell searching behaviour of the subtidal Acadian hermit crab *Pagurus acadianus* (Benedict, 1901). Specifically, we tested the effect of the noise upon the ability of the hermit crabs to orient towards a highly attractive chemical cue, indicative of a newly available shell (Valdes and Laidre, 2018). If anthropogenic noise affects chemically-guided search behaviour, then we predicted that crabs exposed to the noise source would be deterred, despite the chemical signature indicating a highly valuable resource. Given the importance of gastropod shells to hermit crabs, any impairment in normal search behaviour by anthropogenic noise is likely to impact survival and reproductive success.

## RESULTS

**Fig. 1.**
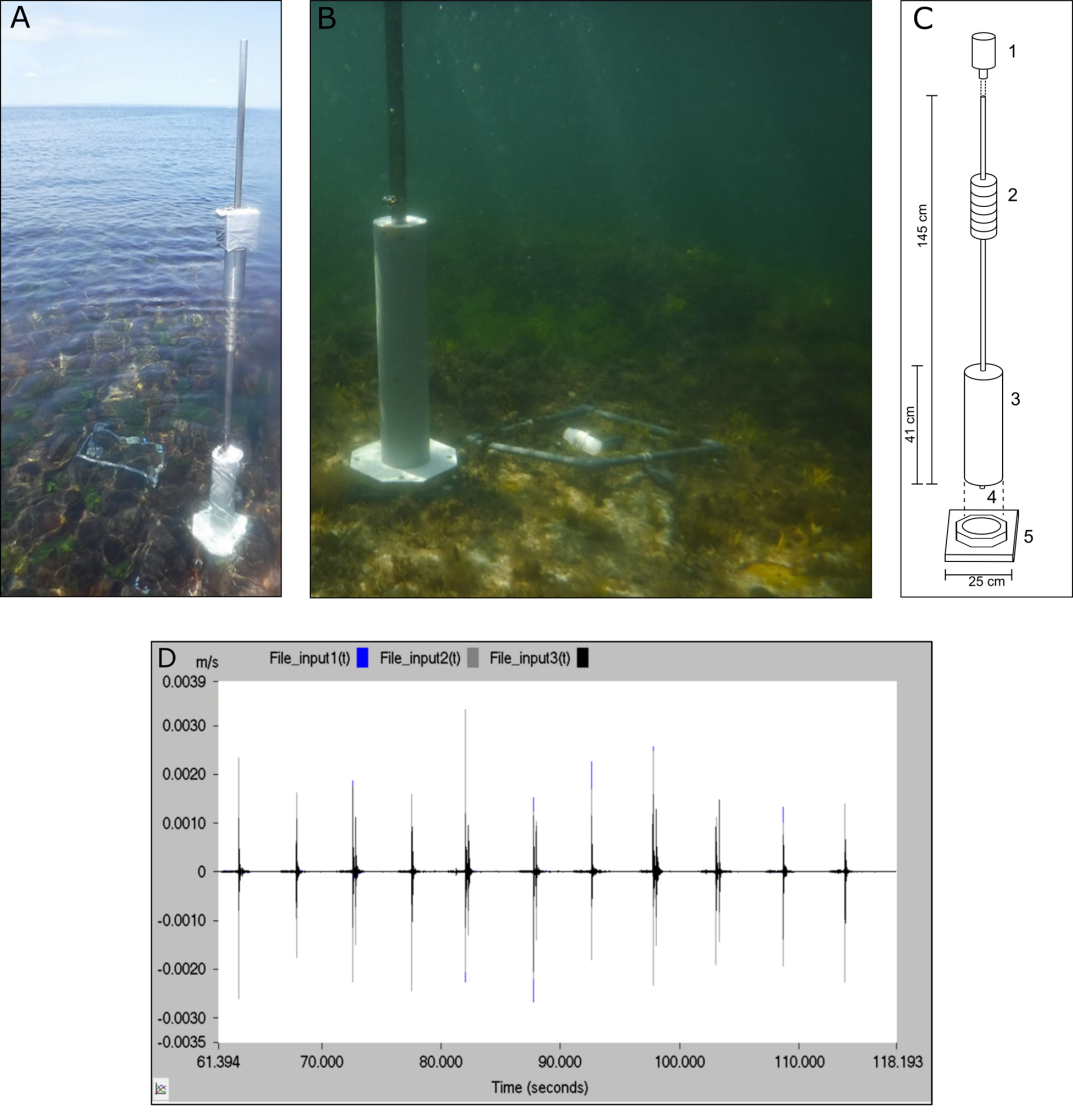
**Experimental apparatus used to create noise in the subtidal.** (A) In each test, a highly attractive chemical cue (indicating a newly available shell home for marine hermit crabs) was placed inside the experimental quadrat. Either noise or silence (control) was then generated in the vicinity. (B) The setup in ∼1 m depth. (C) The setup: 1, hammer weight (∼12 kg); 2, metal stop above the surface; 3, PVC pipe to allow clipping of hammer into base plate; 4, metal bolt allowing pole to directly contact the bedrock; 5, base plate fastened to the bedrock. (D) Example time series of strikes 6 s apart. Ch1–x, Ch2-y, Ch3-z axis, amplitude (peak velocity, m s^−1^).

**Fig. 2.**
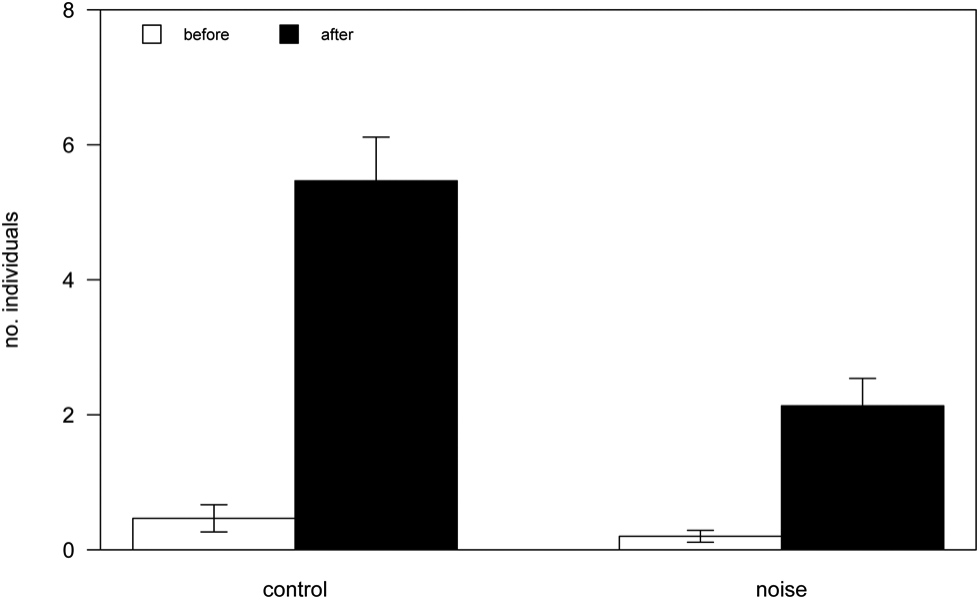
**Number of hermit crabs (mean±s.e.m.) attracted to the chemical cue before and after a 5-min exposure to either a silent control or impulsive noise (*n*=30 for both).**

Treatment (generated noise, Fig. 1, or control) and time (before or after treatment) significantly affected the numbers of crabs (Fig. 2; Table S1). There was a positive relationship between time and the number of crabs present [effect co-efficient, *b=*3.47; standard error of slope, *s.e.*=0.36; *t*-value, *t*(58.00)=9.62; *P*<0.0001], with more crabs present in both post-treatment periods, indicating a general attractiveness of the chemical cue.

There was a negative relationship between treatment and number of crabs, with fewer crabs in the post-noise than the post-control count [*b=*−3.33, *s.e.*=0.56, *t*(112.47)=−5.93; *P*<0.0001; Fig. 2]. There was a significant interaction between treatment and time [*b=*−3.07, *s.e.*=0.72, *t*(58.00)=−4.26; *P*=0.0007], with the difference between control and noise counts being significantly greater in the post-treatment period. As such, the significant main effect of treatment was due to the noise. Indeed, re-centring the time variable over the pre-treatment period indicated the effect of treatment at the pre-treatment period was not reliably different from 0 [*b*=−0.27, *s.e.*=0.56, *t*(112.47)=−0.48; *P*=0.64].

## DISCUSSION

Chemical cues are a widespread form of sensory information in the marine environment, used by animals to locate key resources, avoid predation, detect conspecifics and interpret their surroundings (Breithaupt and Thiel, 2011; Hay, 2009). Being able to use this information is often critical to fitness. Here, we found that an impulsive noise exposure significantly affected the use of chemical information by a species that is common in anthropogenically-impacted coastal areas. Crucially, by testing free-ranging animals in the wild (Laidre, 2018) we allowed individuals to decide which areas to be attracted to or to avoid, providing insight into the impact of noise upon a benthic species in its natural habitat. Our results indicate that anthropogenic noise affects a hard-wired animal search behaviour, implying that individuals will be less successful in finding a new home under noisy conditions.

Like many crustaceans, marine hermit crabs are particularly reliant upon olfaction (Breithaupt and Thiel, 2011; Harzsch and Krieger, 2018) especially within the context of searching for key resources (Rittschof, 1980a; Tricarico et al., 2011). Locating an empty shell upon the seabed is already a complex task, given the rarity of empty shells, the variable number of gastropod predators, the heterogeneity of the physical environment, and sensory barriers (Stevens, 2013), all of which greatly reduce the chances of encountering a new home. Given the importance of the shell to hermit crabs (Laidre, 2011), an impact upon the utilisation of chemical information during shell-searching may have fitness-related consequences. Additionally other chemical cues associated with finding food, interacting with conspecifics, and avoiding predators may also be affected by noise exposure (Acharya, 1998; Hasan et al., 2018; Morris-Drake et al., 2016), which taken together could significantly impact survival. Interestingly, animals may switch to other sensory modalities to compensate for the loss of one modality (Partan, 2017). However, whilst terrestrial hermit crabs are known to use visual and tactile cues as well as chemical cues to locate shells (Laidre, 2010, 2013) these cues may be more limited for marine hermit crabs, given compromised visibility in the water column, lower light levels in the subtidal and complex seabed topography and sedimentation, all of which make the loss of long-range chemical information potentially more impactful.

There is increasing evidence to suggest that anthropogenic noise pollution can have an effect upon responses mediated by other sensory modalities (Acharya, 1998; Chan et al., 2010; de Jong et al., 2018; Hasan et al., 2018; Kunc et al., 2014; Morris-Drake et al., 2016; Walsh et al., 2017), which is known as ‘cross-modal interference’ (Halfwerk and Slabbekoorn, 2015). However, all but one of the aforementioned studies are laboratory-based (Morris-Drake et al., 2016). One such lab-based study (Walsh et al., 2017) found that the shell investigation process (e.g. investigation time, decision-making) of marine hermit crabs was significantly reduced by white noise (measured in terms of water-borne sound). Of the three previous studies relating specifically to olfaction, noise exposure has been shown to significantly affect the time taken to detect and respond to a predator-associated chemical cue (Acharya, 1998; Hasan et al., 2018; Morris-Drake et al., 2016). Noise may also have an effect on visually-guided behaviour used in courtship and conspecific interactions, for example in common cuttlefish and painted gobies (de Jong et al., 2018; Kunc et al., 2014). Further studies indicate reduced response latencies to visual stimuli in noise playbacks (Chan et al., 2010; Simpson et al., 2015, 2016), but in these cases there is a possibility that the stimuli itself consisted of multiple modes. Chan et al. (2010), for example, found that terrestrial hermit crabs modified their anti-predator response to a simulated predatory cue during air-borne sound (boat noise), allowing the predator to get closer. However, it is of note that, unlike marine hermit crabs, terrestrial hermit crabs have limited attraction to chemical cues such as those of gastropod death (Valdes and Laidre, 2019), instead relying on conspecifics for architecturally remodelled shells, which have radically different physical properties (Laidre et al., 2012), altered consequences for survival (Laidre, 2012) and impacts on social dynamics across generations (Laidre, 2019).

Taken together with the results of the current work, there is accumulating evidence to suggest that noise pollution can affect the use of cues and signals cross-modally. Interestingly, other forms of anthropogenic pollution, such as light, may also act cross-modally, for example affecting behaviours guided by sound (Minnaar et al., 2014). This is of particular interest given that anthropogenic sources in the marine environment are rarely emitting energy in a single sensory mode, but are multi-modal (Halfwerk and Slabbekoorn, 2015). Offshore construction operations, for example, may emit heat, light, sound and chemical pollution simultaneously. The result then from one activity may have a complex web of effects, both within and between sensory modes, and at a range of scales.

There are a number of potential mechanisms that may explain how noise affects an animal sensing a cue: (1) noise may mask the cue, such as auditory masking in fish (Hawkins and Chapman, 1975); (2) noise may increase stress, which may lead to physiological changes or behavioural changes (Smith et al., 2004); (3) noise may cause physical damage, affecting the underlying sensory mechanisms used to detect the cue (McCauley et al., 2003); or (4) noise may distract the animal and shift attention (Chan et al., 2010; Dukas, 2004). Further experiments are required to fully understand the mechanism here. However, since some hermit crabs still attended to the chemical cue during noise exposure, despite the exposure level being well within detection capabilities (Roberts et al., 2016), it may be that motivation plays a role here (Chan et al., 2010; Dukas, 2004; Elwood, 1995; Hasan et al., 2018). Crabs carrying a poorer quality shell, being especially in need of acquiring a new home, could be more motivated to orientate towards the chemical cue despite the noise. To test whether this is the case, a shell inadequacy metric could be calculated (Valdes and Laidre, 2018) for crabs that are attracted versus not attracted both in the presence and in absence of noise. Whilst motivation seems like a plausible explanation here, we cannot rule out stress or physical damage, which future work could test by taking haemolymph or tissue samples.

Impulsive sounds, such as those from explosions, pile-driving or seismic surveys, are characterised by a short rise-time (the time to the maximum sound level), and a sharp post-peak decrease, with predominant energy <500 Hz, and with core energy typically below <200 Hz. Key considerations of this type of source are sudden high pressure and particle motion peaks, which may increase the likelihood of sensory damage, and the highly repetitive nature of some of these sources, which may contribute to overall background levels of sound (Popper et al., 2014). Here, the peak energy (in the substrate) of our source was <400 Hz, with a 5 s gap between pulses. Piles during construction are struck repetitively, typically with a very short gap between pulses (e.g. 1–2 s), as here, although our own setup did not actually penetrate the substrate. Our source is therefore much akin to pile-driving, being low frequency, highly repetitive and with a short rise-time and strike duration (Bruns et al., 2016). We aimed to mimic the amplitudes found at a distance from actual construction events rather than right next to a source, and a comparison to the few accessible measurements of seabed vibrations indicates that this is the case (collated within Roberts et al., 2016). That we see effects here at this relatively low amplitude and short exposure duration is of great interest, since peak velocities near actual sources will be significantly greater, and offshore operations last much longer (e.g. days and weeks) than the exposure time here. Indeed, given that chemical cues degrade temporally (Breithaupt and Thiel, 2011), the window of opportunity for detection is small, and so multiple cues could be missed within one anthropogenic construction event.

There are very few field-based experiments testing invertebrates exposed to impulsive noise sources. Indeed, a recent review regarding the effects of impulsive sound listed 33 invertebrate studies, with only seven of these exposing animals to realistic sound levels (Carroll et al., 2017). However, recent experiments suggest physical damage, mortality, behavioural and physiological change in scallops and lobsters exposed to airguns (Day et al., 2016, 2017; Fitzgibbon et al., 2017).

There have been many calls to measure noise stimuli in terms of the actual component which invertebrates detect, particle motion, rather than pressure (most recently Popper and Hawkins, 2018). Since the detection abilities of hermit crabs to water-borne particle motion are largely unknown, we designed an experiment to test the effect of substrate-borne vibration, a stimulus which hermit crabs are definitively sensitive to both in the laboratory (Roberts et al., 2016) and in field experiments (Roberts and Laidre, 2019). Whilst the strikes on the substrate in the present study had an acoustic component, there is no evidence to suggest pressure detection in marine crustaceans to date (Popper et al., 2001), and the measurement of water-borne particle motion is still hindered by a lack of readily-available sensors (Popper and Hawkins, 2018). Although the vast majority of invertebrates are either solely or partially benthic or have benthic life-stages, seabed vibration is not routinely measured (Popper and Hawkins, 2018; Roberts and Elliott, 2017) despite sensitivities to this energy (Roberts et al., 2016), and recent modelling of particle motion waves travelling in the sediment surface (Hazelwood et al., 2018). Indeed, there are only a handful of papers (Day et al., 2016, 2017; Miller, 2015; Roberts et al., 2016, 2017) which include vibration measurement or modelling relating to animal exposure. Of these, the current work is the first to expose a wild free-ranging invertebrate to a source quantified in terms of seabed vibration. Given our findings, it is noteworthy that when assessing the impact of anthropogenic activities upon marine organisms, no regulatory consideration is given to seabed vibration (Popper and Hawkins, 2018; Roberts and Elliott, 2017).

Here we demonstrate for the first time in the marine environment that vibroacoustic noise alters chemical-mediated responses of a free-ranging invertebrate. The results indicate that the impacts of noise pollution should be investigated across sensory modalities, rather than uni-modally, and also indicate that the chemical-mediated responses, which are of great importance to marine animals, must not be overlooked. Similarly, by highlighting the effect of vibration upon a benthic animal, we aim to promote the importance of vibration within the seabed – literally the shaking of the ground – to researchers interested in understanding the impacts of noise pollution on the benthos.

## MATERIALS AND METHODS

### Study site and species

Experiments were undertaken on Appledore Island, within the Isles of Shoals, Gulf of Maine (42° 59′ 21″ N, 070° 36′ 54″ W), operating from Shoals Marine Laboratory. The site used was north of the ‘Great Tidepool’ on the western side of the island, chosen due to high incidence of the study species (*P. acadianus*), accessibility to the subtidal, and consistently good water visibility. Experiments were undertaken in July and August 2018, on calm days (Beaufort scale <3), typically in slots of 3–4 h, within 2 h of low tide. The timing was chosen to align with a water depth of 0.2–1.2 m which was the most practical range to operate the vibrational stimulus safely whilst snorkelling. On average, tests were undertaken in 0.5–0.75 m of water.

The substrate was heterogeneous, being highly uneven in places with shelves, crevices and largely flat areas, with the rock itself covered with a partial sandy sediment and a dense spongy mat of various algae species (Fig. S1). Hermit crabs wander the seabed in this area, predominantly occupying *Littorina littorea* shells (and less frequently, *Littorina obtusata* and *Nucella lapillus* shells).

### Chemical cue

We used a chemical cue (consisting of *L. littorea* flesh mixed with the digestive enzyme trypsin), which is known to be highly attractive to hermit crabs (Valdes and Laidre, 2018). This chemical cue simulates non-destructive predation on gastropods, in which the flesh of the gastropod is consumed by a predator while the shell is left intact. As such, the resulting peptide products indicate a new home, a resource for which marine hermit crabs are evolutionarily hard-wired to search.

Trypsin (0.001 g±0.0001) was added to gastropod flesh (*L. littorea,* 1 g±0.1 g) and 4 ml of distilled water and mixed inside a 20 ml opaque plastic bottle. A fine mesh was secured on the top of the bottle with a rubber band, allowing the chemical cue to disperse once the lid was removed, while still retaining the physical contents. Each bottle had a 170 g fishing weight attached to anchor it to the substrate of the experimental quadrat (Fig. S1). Commercial trypsin derived from bovine pancreas was used for the experiments (Sigma-Aldrich, # T8003; Type I, ∼10,000 BAEE units/mg protein). Gastropod flesh was obtained fresh from live gastropods collected in the local area, with animals dispatched as in Valdes and Laidre (2018) to mimic a predator. Chemical cues were prepared 0.5–1.5 h before the start of the experiments.

### Noise production and measurements

An adapted-for-purpose underwater slide hammer was used to create vibration within the seabed, producing a fully controllable manually-operated stimulus. The stainless-steel pole of the slide hammer had a PVC pipe attached at one end, the diameter of which matched the internal diameter of a base plate fastened to the substrate (Fig. S1), allowing the pole to slot into a free-standing vertical position (Fig. 1A–C). The tip of the pole protruded slightly from the end of the PVC pipe, and had an additional metal piece to accentuate the protrusion, allowing the tip of the pole to contact the rock directly. A single weight consisting of the original hammer plus additional weight (total ∼12 kg) was used as the impactor. At five sites, spaced at 7–12 m (x¯=8.28 m) intervals along 32 m of the shore line, base-plates were bolted onto the substrate after drilling into the bedrock to a depth of ∼8 cm with an underwater pneumatic drill (generator powered, operated from a rigid-inflatable boat). Each base-plate (25×25 cm, 3.5 cm height) consisted of two layers of plastic-coated wood with a central hole (∼5 cm), (Fig. 1A–C, Fig. S1).

When the weight was dropped from the top of the steel pole, it fell vertically and impacted the stop of the hammer halfway down, which elicited vibration down the pole and directly into the rocky substrate it contacted. One drop of the weight created a pulse of vibration similar to a pile-driving ‘strike’ (Fig. 1D, Fig. S2). The dropping of the weight did not create any surface water movement, since the stop of the hammer was above the water line. Each noise exposure consisted of 5 min hammering, with strikes spaced at 5 s intervals. The control treatment consisted of the same arm movements necessary for hammer operation, but without vibration being produced. The position of each test (base plates 1–5) and the exposure (noise or control) were chosen at random, with minor adjustments *in situ* if water depth was impractical. The experimenter had to be present in order to operate the hammer and stood stationary on the substrate next to the hammer in all exposures.

A waterproof tri-axial geophone system (Sensor Nederland, SM-7 375 ohm, IO, sensitivity 28.8 V/m/s) connected to a PHOTON+ Brüel & Kjær Data Acquisition system (Dynamic Signal Analyzer, 3CH), and a rugged laptop (Dell Latitude 14 Rugged 5414) was used to measure vibration. RT Pro software (Brüel & Kjær, v 7.4) was used for live signal observation and post-processing. Measurements on land indicated that the strikes were approximately consistent in amplitude, hence measurements were not taken during each exposure. Instead, a calibration day was undertaken, where measurements were taken at 1 m intervals from the source (0–5 m) in approximately 0.2–0.5 m of water at one of the base plates. Velocity of the strikes (zero to peak, m s^−1^) was calculated by averaging across 10 strikes per axis of vibrational motion. Spectra of individual strikes were calculated in RT Pro (Hanning window, FFT magnitude 1024).

Noise events consisted of repetitive low-frequency pulses, with peak energy in all three axes at 60 Hz, and in the vertical axis at 400 Hz, with the bulk of the energy at 500–700 Hz (Fig. S3). The time history profile was similar to that of pile driving, having a short rise-time <0.015 s (the time from the start of the pulse to the peak velocity) and strike duration (∼0.1 s), as compared to other signals (Bruns et al., 2016; Hawkins et al., 2014) (Fig. S3). The spectra of pile driving in terms of sediment vibration would be expected to have bulk energy between 5–50 Hz (Bruns et al., 2016), with core energy <500 Hz, (Fig. S3). The average peak velocity, measured at 1 m, was 0.0005 m s^−1^, 0.00001 m s^−1^ and 0.0001 m s^−1^ for CH1, 2 and 3 (specifically in the order of vertical first: y, x, z plane) respectively, dropping to 0.00009 m s^−1^, 0.00002 m s^−1^ and 0.00002 m s^−1^ at 5 m. As such, the stimulus was greater than the known lowest threshold of detection ability (determined behaviourally) in the range of 5–500 Hz (Roberts et al., 2016).

### Experimental procedure

All experiments were undertaken in the field so that hermit crabs could range freely throughout the wider area, ‘choosing’ which areas to be attracted to or to avoid. The sequence of each test consisted of the following procedure: the slide hammer was fastened into one of the randomly assigned base-plates (1–5), and a weighted quadrat (30×30 cm) was placed on the substrate next to the setup. The experimenter then stood stationary next to the hammer, in position for the test, for a 5-min acclimation period to allow crabs in the immediate vicinity to return to normal activities. At the onset of the test, the number of hermit crabs inside the quadrat was counted (‘before’ count) and the test bottle was placed on the substrate with the lid subsequently removed. This allowed the chemical cue to disperse from the bottle into the water column. The quadrat was then immediately exposed to 5 min of noise or silent control, randomly assigned. After 5 min the number of hermit crabs in the quadrat was counted (‘after’ count). An exposure period of 5 min was chosen since previous work using the same chemical cue showed rapid peaks in hermit crab attraction followed by a decline after 5 min (Valdes and Laidre, 2018). Experiments were undertaken across 5 days, with a total of *n*=60 tests (*n*=30 for noise and *n*=30 for the control).

### Statistical methods

All graphical representations and statistical tests were undertaken in RStudio v.1.1.453 (RStudio, 2015), with use of the lme4 (Bates et al., 2015) and the lmerTest package (Kuznetsova et al., 2017). Linear mixed effect analysis (fit using restricted maximum likelihood and degrees of freedom estimated using Satterthwaite's method) was used to look at the effect of treatment, time, and their interaction on the number of crabs, with fixed effects of treatment (noise coded as 0.5 and control coded as −0.5) and time (the time of the count, pre- treatment coded as −1 and post-treatment coded as 0). The time variable was coded so the main effect of treatment could be interpreted after the treatment was applied. The model also included a random intercept for station, defined as the specific incidence of the count (numbered arbitrarily from 1–60). Visual inspection of the residual plots did not reveal strong deviations from homoscedasticity or normality.

## Supplementary Material

Supplementary information
