# Supplementary material for: Finding a home in the noise: cross-modal impact of anthropogenic vibration on animal search behaviour
Source: Biol Open. 2019 Jul 15;8(7):bio041988. doi: 10.1242/bio.041988 (PMC6679394; doi:10.1242/bio.041988)
Supplement: Supplementary information [file biolopen-8-041988-s1.pdf]

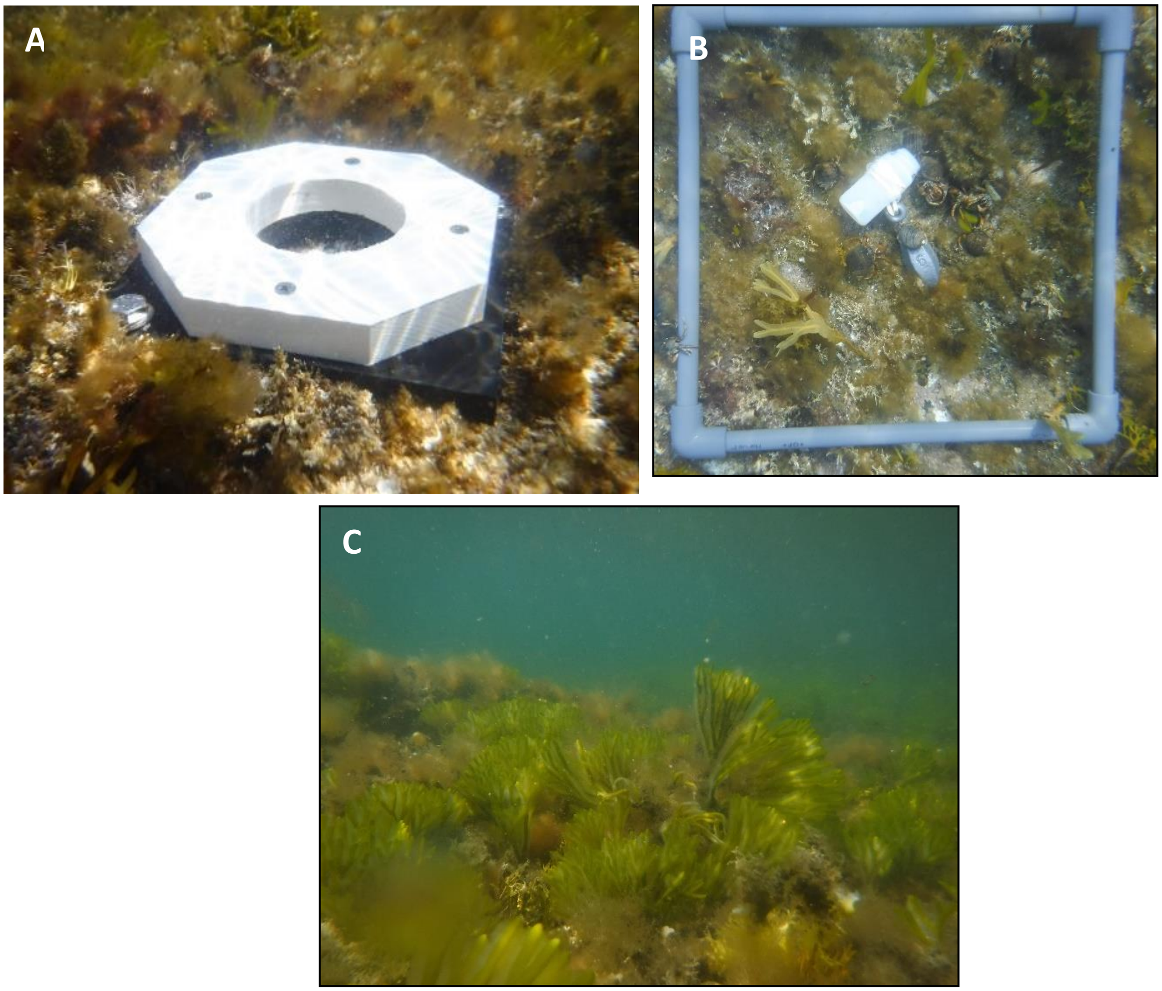

Figure S1. A. Five octagonal base plates (25 x 25 cm, 3.5 cm height), each with a circular cut-out (5 cm diameter) central area were bolted onto the bedrock to allow the adapted slide hammer to be clipped in and out. The base plates were spread randomly along 32 m of subtidal, with c.a. 8 m space in between each one; **B.** A plastic bottle, anchored by a small fishing weight, contained the chemical cue and was placed inside the experimental quadrat; **C.** Typical conditions in the experimental area, consisting of uneven rocky terrain covered with a dense spongey mat of algae.

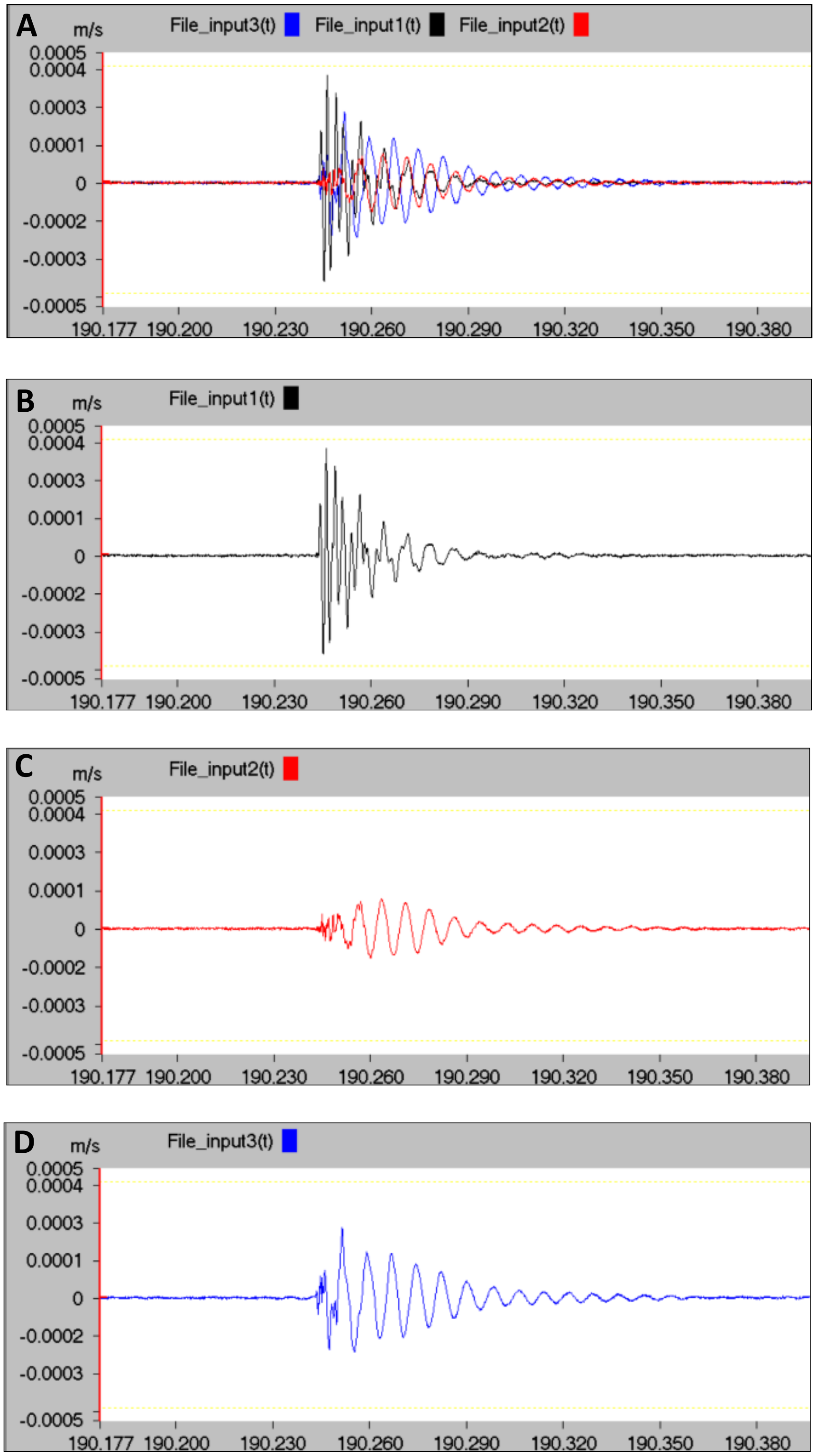

Figure S2. A. Example waveform of one strike of the hammer in all axes together; B - D the same strike split by plane of motion (y, x, z), (zero-peak, m s^{-1}).

Biology Open • Supplementary information

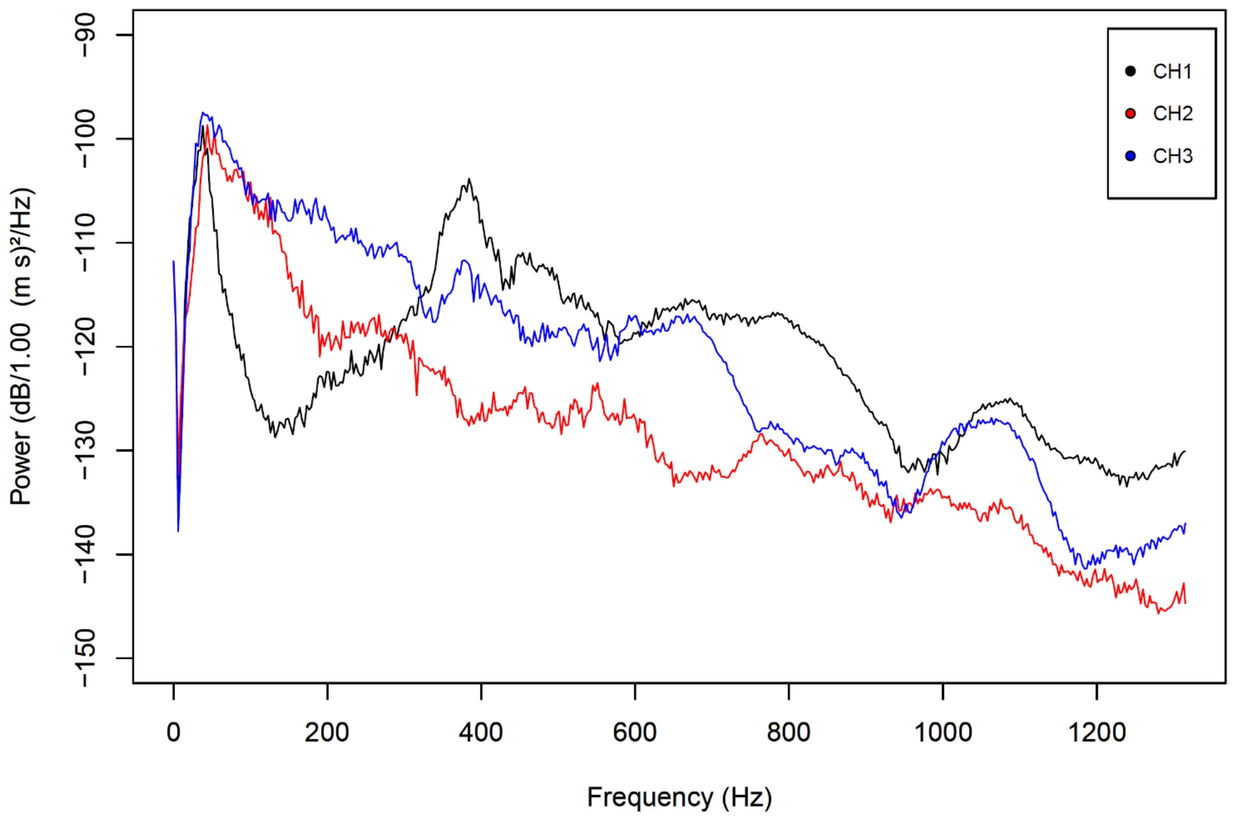

Figure S3. Spectra of the noise exposure (average power spectra (dB/(m/s^2)/Hz; Hanning, 1024 pts; dBref 1 = 0 dB) across 10 strikes for each axis (consisting of a repetitive impulsive signal. CH1 vertical plane, the other two planes are horizontal.

Biology Open • Supplementary information

Table S1. Result of the linear mixed effect model with interaction effect; with dependent variable (no.crabs, the counts of crabs); fixed effects of time (time of the count, before or after treatment), treatment (noise or control), and random effect of station (being the specific instance when/where the count took place).

	no. crabs
Time	3.4667***
(S.E)	(0.3603)
Treatment	-3.3333***
	(0.5618)
Time : Treatment	-3.0667***
	(0.7207)
Constant	3.800***
	(0.2809)
Station (random effect) N total	60
Var. (Std. dev.)	0.8391 (0.916)
Observations	120
Log likelihood	-260.6
Akaike Inf. Crit.	533.1
Bayesian Inf. Crit.	549.9

Note: *p<0.05; **p<0.01; ***p<0.001
